# In Vitro Fertilization in a Nulliparous Female Resulting in Placenta Increta and Postpartum Hemorrhage

**DOI:** 10.7759/cureus.18042

**Published:** 2021-09-17

**Authors:** Juliette M Kassas, Lauren M Blue, Carol A Brenner

**Affiliations:** 1 Osteopathic Medicine, University of New England, Biddeford, USA; 2 Obstetrics and Gynecology, Speare Memorial Hospital, Plymouth, USA

**Keywords:** placenta accreta spectrum, placenta increta, female infertility, peripartum hysterectomy, in vitro fertilization (ivf)

## Abstract

A 32-year-old female with unexplained infertility delivered a healthy male infant at 39 weeks 0 days gestational age; the pregnancy was facilitated by in vitro fertilization. Shortly after delivery, she was found to have a morbidly adherent placenta. Attempted removal resulted in postpartum hemorrhage and ultimately hysterectomy after attempting multiple fertility preserving methods to achieve hemostatic control. Pathology results revealed a diagnosis of a 0.1 cm placenta increta (Grade 2 placental villi invasion), the least common diagnosis within the placenta accreta spectrum (PAS). Likely due to the small point of trophoblastic invasion, the diagnosis and outcome were not foreseen. This case highlights the need for additional data collection and development of standardized guidelines for the diagnosis and management of PAS, given a patient’s risk factors. Current research may be limited by stigmatization surrounding infertility and reproductive-altering surgeries (e.g. hysterectomy). Additionally, counseling in all stages of pregnancy is critical to achieving the best patient-centered outcomes.

## Introduction

During the third stage of delivery, the placenta, which supplies nutrients and gas exchange to the developing fetus, spontaneously detaches from the uterus and is subsequently expelled or removed. In abnormal circumstances, the placenta may adhere to the uterus because placental villi (trophoblasts) have grown into one or more layers of the uterus. It is commonly theorized that this pathologic process most commonly occurs in weakened areas of the uterine endometrium. Insufficient point attachment and nutritional exchange to support growth and maintenance of the placenta and fetus may encourage further growth into the uterine wall [1,2]. This phenomenon occurs in 0.2% of pregnancies [3]. The International Federation of Gynecology and Obstetrics (FIGO) has defined placental villous growth into the uterine wall as placenta accreta spectrum (PAS), which is comprised of three subtypes: placenta adherenta or creta, placenta increta, and placenta percreta [4]. Placenta adherenta or creta (Grade 1 PAS) is defined as a villous attachment to the superficial myometrium and occurs in 63% of cases [3-5]. Placenta increta (Grade 2 PAS) is defined as a villous invasion into the myometrium and is the least common of the subtypes, diagnosed in 15% of cases [3-5]. Finally, placenta percreta (Grade 3 PAS) is defined as villous tissue within or breaching the serosal layer; it occurs in 22% of cases [3-5].

The most significant risk factors for the development of PAS include but are not limited to uterine surgery, placenta previa after cesarean delivery, maternal age >35 years, multiparity, history of irradiation to the pelvis, and infertility (including infertility procedures) [6-10]. One observational study suggests that 80% of patients with PAS have a history of uterine surgery, which includes cesarean delivery, curettage, and myomectomy [1]. The condition is becoming more prevalent due to the increased popularity of cesarean deliveries [6,7].

There are not any definitive methods for diagnosing PAS prior to the end of pregnancy, although it may be detected incidentally through imaging studies [11]. Manual extraction of the placenta is often attempted once determined that spontaneous expulsion is unlikely. In the setting of PAS, attempted removal of the placenta provokes significant bleeding, often resulting in hemorrhage. Due to the significant risk of hemorrhage and its inherent morbidity, further research on a standardized approach for detection and management of PAS is warranted [12].

## Case presentation

A 32-year-old Caucasian gravida 1 para 0 (G1P0) woman without any significant past medical or surgical history was followed by our department throughout the duration of her pregnancy, which was facilitated by in vitro fertilization. Prior to pregnancy, the patient had regular menstrual periods and cycles lasting 30-32 days on average with a variability of +/- 5 days. Papanicolaou (Pap) smear conducted 5 years prior to pregnancy revealed a low-grade squamous intraepithelial lesion that was negative for human papillomavirus (HPV). A subsequent Pap smear conducted 2 years prior to pregnancy was negative for abnormality. She denied a history of pelvic inflammatory disease (PID), sexually transmitted infections (STIs), tubo-ovarian abscess, colitis, appendicitis, and endometriosis. Despite not having any chronic medical conditions, being a non-smoker, and having a healthy, active lifestyle, the patient had a history of unexplained infertility for which she underwent an extensive medical workup and multiple treatment modalities.

**Figure 1:**
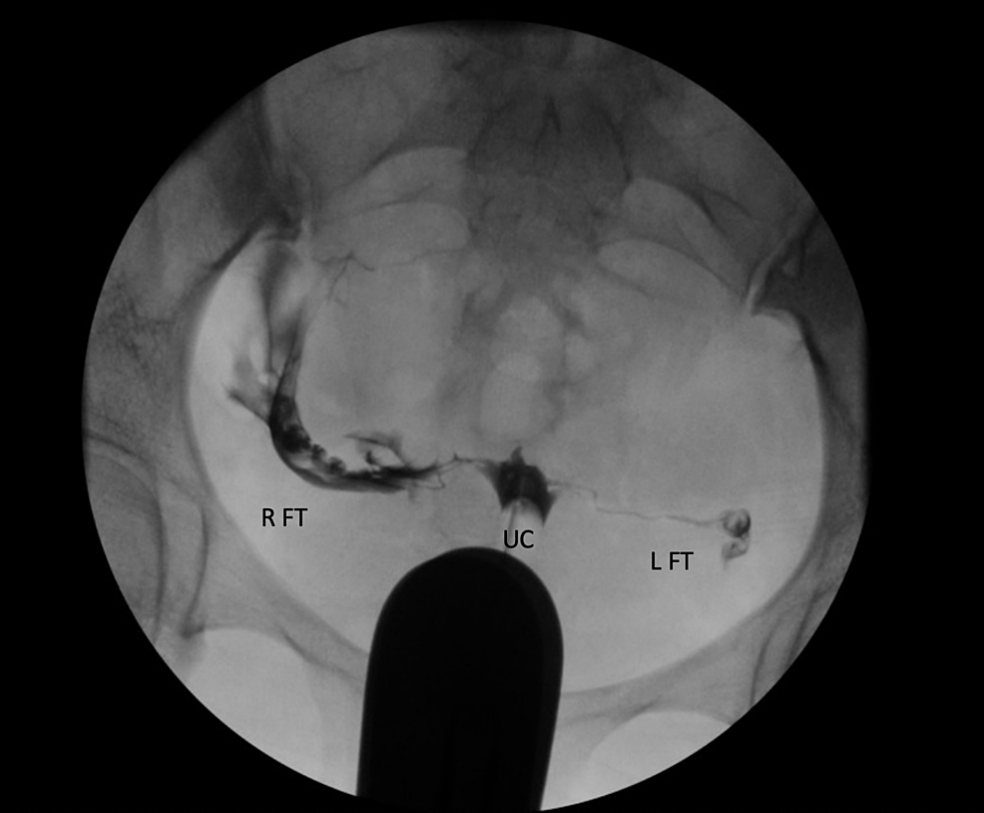
Hysterosalpingogram This figure shows contrast solution traveling through the right fallopian tube (R FT), uterine cavity (UC), and left fallopian tube (L FT).

The patient previously used an intrauterine device for contraception, which was removed approximately 4 years prior to this pregnancy. Approximately 2 years prior to the start of pregnancy at her routine gynecologic exam (during which she had her second Pap smear, as noted previously), the patient stated that she was trying to become pregnant. Two weeks prior to this exam, her male partner had an overall normal semen analysis. The patient at this time was provided with prenatal guidance regarding timed intercourse and anticipatory guidance. She was also instructed to take daily prenatal vitamins and docosahexaenoic acid (DHA). Roughly 4 months after this appointment (at this point 8-9 months of actively trying to become pregnant) the patient sought out additional procreative guidance after unsuccessfully conceiving. The patient was advised to use an ovulation indicator, continue timed intercourse, and maintain healthy lifestyle habits. Laboratory testing was also conducted, and the following values were reported at this time: dehydroepiandrosterone (DHEA) 7.35 ng/mL, estradiol 48 pg/mL, progesterone 0.26 ng/mL, testosterone 21 ng/dL, sex hormone-binding globulin 73 nmol/L, free testosterone 2.1 pg/mL, follicle-stimulating hormone (FSH) 6.1 mIU/mL, prolactin 15.04 ng/mL, luteinizing hormone (LH) 4.35 mIU/mL, LH:FSH 0.7. These values were all considered to be within normal limits for being in the follicular phase of the menstrual cycle. These results were followed by a hysterosalpingogram to assess potential structural abnormalities, which was conducted 1 month later and shown in Figure 1. The impression noted a widely patent right fallopian tube and a patent left fallopian tube. Because of the widely patent right side, the contrast flowed very quickly, and it was difficult to appreciate the detail of the endometrial cavity. Overall, no focal abnormalities were noted. The patient subsequently underwent three cycles of clomiphene at a 50 mg dose for 5 days (instructed to be used during days 5-9 of the menstrual cycle) and one cycle of clomiphene at 200 mg with the same regimen. She was unsuccessful at conceiving after a combined four cycles and experienced gastrointestinal-related side effects with the 200 mg dose. She was then unsuccessfully trialed on letrozole 2.5 mg for 5 days using the same regimen and was referred to an infertility clinic.

The patient was next managed by our network 8 weeks into her pregnancy in the emergency department with a chief complaint of vaginal bleeding with abdominal cramping. She stated to the provider that she had undergone in vitro fertilization with the implantation of two 5-day-old embryos. A transvaginal ultrasound confirmed a singleton live intrauterine pregnancy with an estimated age of 8 weeks 0 days. She was subsequently discharged home.

The patient was reintroduced to our department during her first prenatal visit at 10 weeks 4 days gestational age (GA). At this appointment, she was screened and found negative for the following conditions: asymptomatic urinary tract infection, chlamydia, gonorrhea, hepatitis c, hepatitis b, human immunodeficiency virus (HIV), and syphilis. She was also found to be rubella immune with a negative urine drug screen. Her blood type was confirmed as B positive with a negative antibody screen. The patient elected to have genetic testing at this point in time which revealed an expected representation of chromosomes 21, 18, and 13 (normal result). Y chromosomal material was also detected, consistent with a male fetus. The patient continued with an uneventful pregnancy with a normal fetal anatomy scan at 20 weeks. Her 1-hour glucose tolerance test was borderline at 143 mg/dL and followed up with a 3-hour glucose tolerance test that revealed fasting, 1-hour, 2-hour, and 3-hour results of 84, 146, 111, and 102 mg/dL, respectively. The patient was swabbed for Group B Streptococcus (GBS) in her 36th week of pregnancy and was found to be colonized (positive).

The patient presented at 39 weeks and 0 days GA at the labor and delivery unit in active labor claiming rupture of membranes with clear fluid within the last 3-4 hours. Shortly after admission, she was adequately treated for the GBS colonization and provided epidural analgesia. She progressed through labor without augmentation and had a category 1 fetal heart tracing. After complete dilation of the cervix and more than 3 hours of active pushing, the course of labor was augmented by uncomplicated vacuum assistance. This resulted in the delivery of a healthy male infant and a first-degree laceration. After 30 minutes, the placenta had not detached from the uterus with the application of gentle traction. The patient had consented at this point in time for manual removal of the placenta, which was found to be morbidly adherent to the uterus. The attempt of placental removal resulted in atony of the uterus and brisk vaginal bleeding. Bimanual uterine massage and uterotonic drugs (oxytocin**, **methylergometrine, and carboprost) were sequentially administered and were unsuccessful at controlling the bleeding. The patient was urgently brought into the operating room for further management. First, a Bakri balloon tamponade catheter was placed without improvement, and the decision was made to move on to exploratory laparotomy. After transitioning to an abdominal procedure, the physician placed bilateral O’Leary sutures followed by multiple longitudinal and box-type compression sutures in the myometrium of the uterus. The bleeding remained uncontrolled and the B-Lynch technique was subsequently attempted. This sequence was used in an effort to preserve the patient's fertility.

Ultimately, a peripartum hysterectomy was needed despite following the uterotonic algorithm. A massive transfusion protocol was also declared, requiring the administration of six units of packed red blood cells (RBCs), two units of fresh frozen plasma, and two units of platelets in order to achieve hemodynamic stability. The patient was discharged after a 3-day hospital stay. In follow-up, her pathology report revealed a diagnosis of a 0.1 cm placenta increta. Placenta images are shown inFigure 2 and Figure 3.

**Figure 2:**
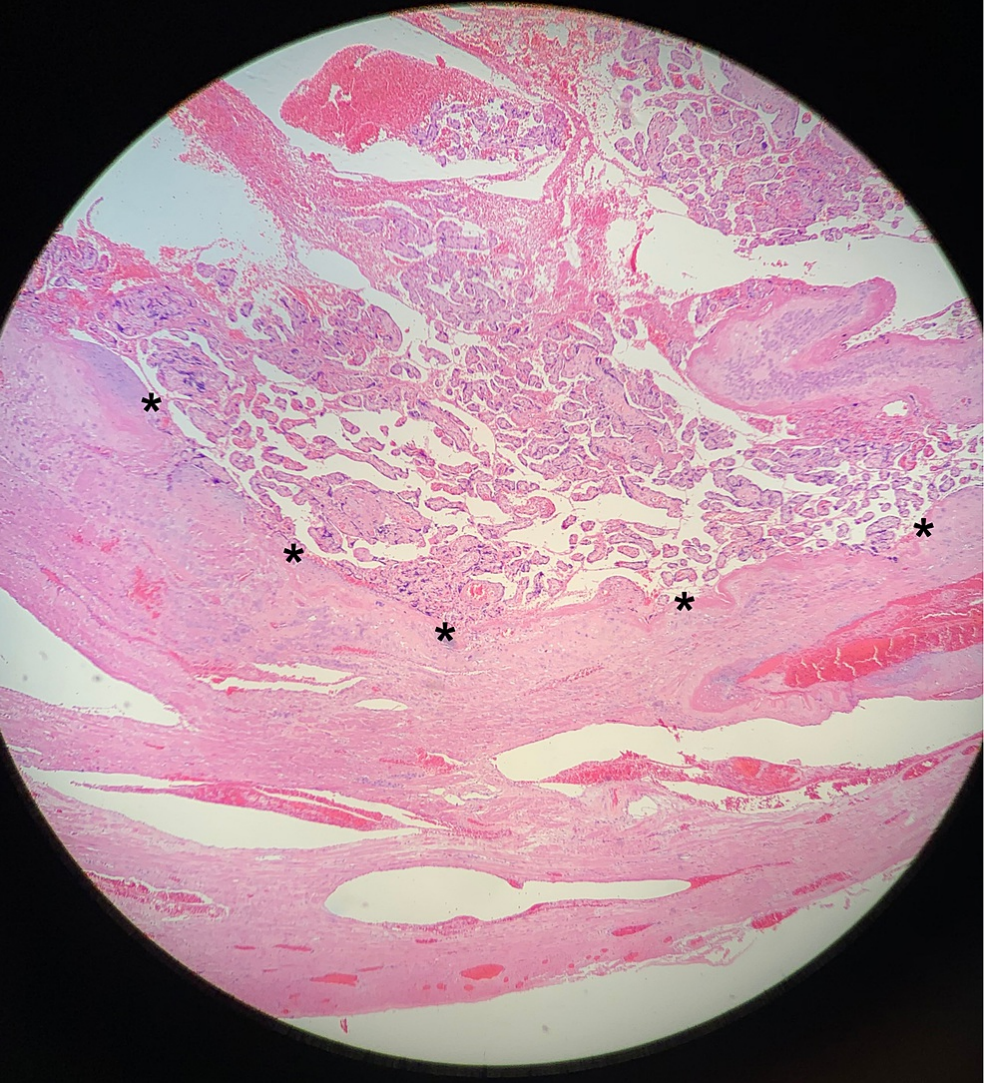
Normal Placentation This image depicts a normal representation of placental attachment to the uterus. The symbols (*) demonstrate the separation of the placenta (top) from the uterine wall (bottom). (H&E 400X)

**Figure 3:**
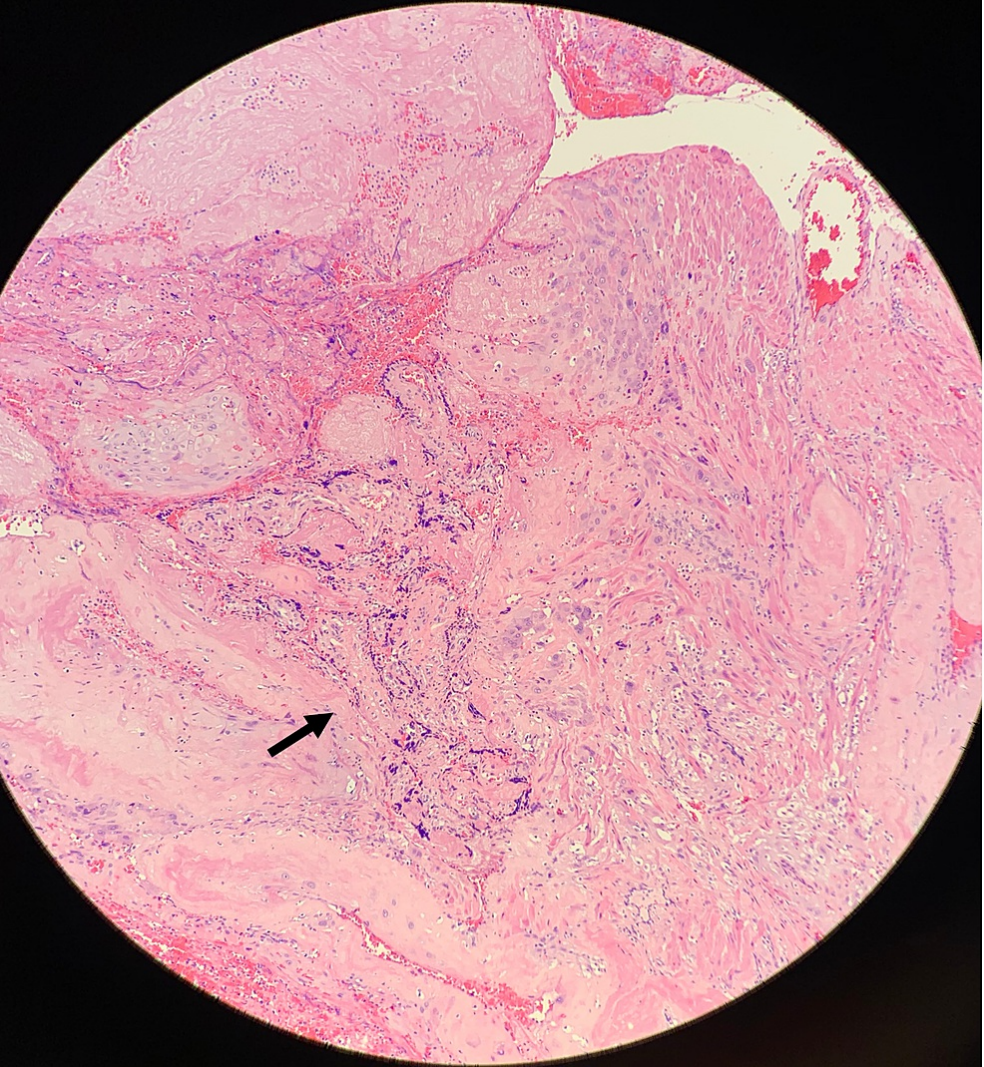
Placenta Increta In contrast to Figure 2, this image shows placental invasion into both the endometrial and myometrial layers of the uterus. The invasion depth is best represented by the black arrow. (H&E 400X)

## Discussion

Due to low incidence, PAS is not commonly screened for without significant risk factors (predominantly prior history cesarean section or other uterine surgery). Although screening for this condition has been proven to be linked to improved maternal and fetal outcomes, there are no definitive guidelines to suggest when it is necessary or recommended [12]. It is noted in the literature that 18% of women with a morbidly adherent placenta are nulliparous, suggesting that risk is difficult to determine [13]. This may be a contributing factor to the ambiguity behind the screening recommendations. At present, screening is left to the discretion of the clinician.

PAS may be detected incidentally on obstetric ultrasound examination. Common ultrasonographic findings which are suggestive of this diagnosis include placental lacunae, disruption of the bladder lining, thinning of the myometrium, and vascular irregularities, among others [14-16]. In one small study of seven patients, a gestational sac in the lower uterine segment with a history of previous cesarean delivery suggested the possibility of placenta accreta [17]. Placental growth over previous cesarean scar has also been used as a predictor for the outcome of PAS [18]. Identification of these findings may be helpful in the development of management guidelines. Additionally, detection in the future with the option of more advanced imaging technology and/or prior identification could allow for alternative safer delivery method options and preparation for appropriate resources at delivery [19]. Avoiding adherent placental removal would likely be advised and would eliminate the dangerous risk of PPH [19]. In order to preserve fertility, conservative measures are recommended before drastic procedures. Uterotonic medicines, intrauterine packing (Bakri balloon), external compression with uterine sutures (B-Lynch), and selective devascularization by ligation or embolization of the uterine artery are all conservative alternatives for PPH [20]. 

In this patient's case, the probability of PAS may have not been considered because she was nulliparous, healthy, and without major risk factors other than facilitation of pregnancy through in vitro fertilization. This modality of achieving pregnancy is associated with a higher risk of PAS compared to the general population (1.6% versus 0.12% in one study), but it is still relatively uncommon [9]. Because the villous invasion was incredibly small, measured 0.1 cm by pathology report, it is very unlikely that it would be visualized on imaging studies. It should be noted that complete or focal adherence of the placenta to the uterus is associated with massive PPH. The size and depth of invasion do not affect the result [9]. Overall, this outcome with minuscule risk highlights the importance and necessity to counsel patients about the risks and options that surround pregnancy, no matter the likeliness of the outcome. This patient had an uncomplicated pregnancy and was considered a low-risk delivery. Shortly after becoming a mother, she battled for her life and lost her fertility in the process. Counseling is especially important for patients who utilize assisted reproductive technology (ART), which includes ovulation stimulation medications, intrauterine insemination (IUI), and in vitro fertilization (IVF), among others. One study claims that ART, no matter the modality, is associated with an increased risk of several adverse obstetric and perinatal outcomes including placenta previa, preterm delivery, and low birth weight [21].

## Conclusions

PAS is diagnosed in 0.2% of pregnancies resulting in delivery and encompasses three subtypes: placenta adherenta or creta, placenta increta, and placenta percreta, which indicate pathologic placental villous tissue attachment to the superficial myometrium, invasion into the myometrium, or invasion into or beyond the serosa, respectively. The size and depth of invasion of placental tissue do not change the outcome or extent of a PAS pregnancy. Guidelines do not currently exist regarding the screening, detection, or management of PAS. However, it is documented that in vitro fertilization (IVF) is associated with a higher risk of PAS compared to the general population for unknown reasons.
